# Post-COVID-19 Syndrome: Involvement and Interactions between Respiratory, Cardiovascular and Nervous Systems

**DOI:** 10.3390/jcm11030524

**Published:** 2022-01-20

**Authors:** Valeria Visco, Carolina Vitale, Antonella Rispoli, Carmine Izzo, Nicola Virtuoso, Germano Junior Ferruzzi, Mario Santopietro, Americo Melfi, Maria Rosaria Rusciano, Angelantonio Maglio, Paola Di Pietro, Albino Carrizzo, Gennaro Galasso, Alessandro Vatrella, Carmine Vecchione, Michele Ciccarelli

**Affiliations:** 1 Department of Medicine, Surgery and Dentistry, University of Salerno, 84081 Salerno, Italy; valeriavisco1991@libero.it (V.V.); carolinavitale.med@gmail.com (C.V.); antonellarispoli@gmail.com (A.R.); carmine.izzo93@gmail.com (C.I.); germano.ferruzzi.jr@gmail.com (G.J.F.); santopietromario.ms@gmail.com (M.S.); myra80@gmail.com (M.R.R.); amaglio@unisa.it (A.M.); pdipietro@unisa.it (P.D.P.); albino.carrizzo@gmail.com (A.C.); ggalasso@unisa.it (G.G.); avatrella@unisa.it (A.V.); cvecchione@unisa.it (C.V.); 2 Cardiology Unit, University Hospital “San Giovanni di Dio e Ruggi d’Aragona”, 84081 Salerno, Italy; n1virtuoso@gmail.com (N.V.); americo.melfi1@tin.it (A.M.); 3 Vascular Physiopathology Unit, IRCCS Neuromed Mediterranean Neurological Institute, 86077 Pozzilli, Italy

**Keywords:** post-COVID-19 syndrome, long COVID, long-term COVID-19, “long hauler” syndrome, clinical manifestations, sequelae, pandemic

## Abstract

Though the acute effects of SARS-CoV-2 infection have been extensively reported, the long-term effects are less well described. Specifically, while clinicians endure to battle COVID-19, we also need to develop broad strategies to manage post-COVID-19 symptoms and encourage those affected to seek suitable care. This review addresses the possible involvement of the lung, heart and brain in post-viral syndromes and describes suggested management of post-COVID-19 syndrome. Post-COVID-19 respiratory manifestations comprise coughing and shortness of breath. Furthermore, arrhythmias, palpitations, hypotension, increased heart rate, venous thromboembolic diseases, myocarditis and acute heart failure are usual cardiovascular events. Among neurological manifestations, headache, peripheral neuropathy symptoms, memory issues, lack of concentration and sleep disorders are most commonly observed with varying frequencies. Finally, mental health issues affecting mental abilities and mood fluctuations, namely anxiety and depression, are frequently seen. Finally, long COVID is a complex syndrome with protracted heterogeneous symptoms, and patients who experience post-COVID-19 sequelae require personalized treatment as well as ongoing support.

## 1. Introduction

While the acute symptoms of COVID-19 have been extensively reported, the longer-term effects are less well identified, because of the quite short history of the pandemic [1,2]. Specifically, most COVID-19-positive patients recover totally within 3–4 weeks after onset of infection; nevertheless, in some cases, prolonged or recurrent symptoms can be seen even weeks or months after COVID-19 recovery [3,4]. The UK’s Office for National Statistics assessed that one in five patients report symptoms beyond 5 weeks, while 10% have symptoms persevering over 12 weeks [5]. Improving the handling of these patients needs the contextualization and classification of the long-term symptoms [6]. Actually, there are varied nomenclatures and time ranges (3, 4 or 12 weeks) used to explain the condition, inadequate knowledge on its etiology and a lack of evidence for the possible treatments [6]. Indeed, various authors have used different names such as “post-COVID-19 syndrome”, “long COVID-19”, “long-term COVID-19 effects”, “long haulers” and “persistent COVID-19 symptoms” [4], which refer to various conditions such as lasting inflammation, sequelae of organ damage, hospitalization and social isolation [7]. However, the WHO has established a clinical case definition of post COVID-19 syndrome: “it occurs in individuals with a history of probable or confirmed SARS-CoV-2 infection, usually 3 months from the onset of COVID-19 with symptoms and that last for at least 2 months and cannot be explained by an alternative diagnosis” [8].

The original cause of the persistence of symptoms has yet to be recognized, but several hypotheses have been produced [6]: aberrant immune responses, virus-specific pathophysiological alterations, inflammatory damage in response to the acute infection [9] and mechanisms of viral persistence in certain tissues [10,11], SARS-CoV-2 interactions with host microbiome/virome communities, clotting/coagulation issues and dysfunctional brainstem/vagus nerve signaling [12]. Moreover, the roles of exosomes and mast cells [13,14,15] recently came under consideration. Furthermore, underlying risk factors can be involved: severity of early COVID-19, including symptom load, level of hospital care and necessity for mechanical ventilation [5], female gender [5], age [16,17,18,19], presence of comorbidity [18,19,20,21,22] and minority ethnicity [22,23] foster the development of long COVID.

Moreover, COVID-19 vaccines decrease the risk of contracting infection; however, studies disagree on their protective effect against long COVID [24]. Certainly, vaccines reduce the risk of long COVID by lowering the chances of contracting COVID-19 in the first place, however, for patients that do experience the infection, trials suggest that vaccination might only reduce the risk of long COVID, or have no effect on it at all [25]; consequently, long COVID can arise even after an asymptomatic coronavirus infection [24].

The respiratory system is known to be the most frequently affected by the COVID-19 acute illness phase, which is prolonged in the post-COVID-19 phase after patients’ recovery [4]. However, it is now well recognized that extrapulmonary systems such as the cardiovascular (CV) and nervous systems are also affected [4], producing symptoms such as cough, shortness of breath, fatigue, headache, brain fog, chest pains, gastrointestinal issues, joint pains and loss of taste and smell, along with neuropsychiatric symptoms, for instance, insomnia, delirium, depression and anxiety [26,27,28,29,30,31,32,33,34] (Table 1).

Consequently, in this review, we discuss the currently available published literature related to the possible involvement of the lung, heart and brain in post-viral syndromes and their reciprocal crosstalk, and describe the suggested management for post-COVID-19 syndrome.

## 2. Lung Involvement

The respiratory system is the primary target of COVID-19 infection, resulting in a broad spectrum of clinical and radiological manifestations. Although in about 80% of cases the infection is confined to the upper airways, in 20% the virus reaches the alveoli, leading to the formation of pulmonary infiltrates, with the onset of dyspnea, cough and fever, associated with varying degrees of hypoxemia and radiological abnormalities [35]. Interstitial pneumonia is the leading cause of hospitalization in patients with COVID-19. In most cases the disease is mild–moderate, however, progression to severe respiratory failure and acute respiratory distress syndrome (ARDS) occurs in 5–10% [36,37,38]. Among COVID-19 survivors, many patients continue to experience respiratory symptoms, and several studies have reported abnormalities in pulmonary function tests (PFTs) and chest CT images even months after hospital admission. The prevalence of these findings varies from one study to another, depending on the methodological approach and follow-up time [39,40,41]. Dyspnea and cough are the most frequently described respiratory symptoms. The biological mechanisms underlying the persistence of respiratory symptoms are not fully clear, but are probably related to the pathological processes triggered in the acute phase. Persistent endotheliopathy resulting in a pro-coagulant state and inflammatory cytokine production could be involved [40,42].

In a recent meta-analysis of 16 cohort studies with hospitalized patients, with follow-up periods > 1 month post-discharge or >2 months post-admission, the prevalence of abnormalities in lung function was approximately 20%. The most common abnormality observed was diffusion impairment, followed by restrictive ventilatory defects [39].

It is interesting to note that in several studies the presence of respiratory symptoms was not related to functional or radiological alterations. Indeed, in a subgroup of 390 patients of a large prospective cohort study, evaluated after a median of 6 months, no correlation was found between symptoms, lung function, exercise capacity and chest CT imaging. In this study, DLCO and 6-min walk distance were reduced in 29–56% and 24–29% of cases, respectively, and radiological alterations at chest CT scan were present in 41–45% of patients [26]. Moreover, in a study of 134 patients, fatigue and/or dyspnea were present in 30% of patients at 6 months of follow-up; however, these symptoms were not justified by significant abnormal findings in lung function tests or chest CT scans [43]. Furthermore, in a prospective cohort study, which enrolled 103 patients, 54% of patients had persistent dyspnea at the 3-month follow-up visit; however, most patients had lung volumes within the reference limits, while only 24% had reduced DLCO [41]. Chest CT scans showed ground-glass opacities in 25% of patients and parenchymal bands in 19% of patients [41]. However, Cortes-Telles et al. reported that patients with persistent dyspnea had reduced lung volume, lower DLCO and increased exertional desaturation, compared to those without [44]. According to the authors, persistent dyspnea could be explained by greater constraints on tidal volume expansion, exertional hypoxemia and a more rapid and shallow breathing pattern adopted by these patients [44]. The discrepancy between symptoms, lung function and imaging resulting from the studies highlights the necessity of a better understanding of the pathophysiological mechanism underlying this new pathology.

Long-term pulmonary sequelae are of particular interest in critical patients who survive COVID-19. Most published data showed a high prevalence of functional impairment and pulmonary structural abnormalities in patients requiring ICU admission [39,45,46]. Gonzalez et al. evaluated 62 patients admitted to an ICU with ARDS secondary to COVID-19 at the 3-month follow-up. Eighty-two percent of patients had reduced DLCO and 70% had signs of lung damage at CT scan. The length of invasive mechanical ventilation during the ICU stay and age were associated with the severity of radiological alterations [45]. Similar results were reported in 48 mechanically ventilated survivors of COVID-19 3 months after hospital discharge [46]. The growing attention towards these patients is also due to greater risk of developing pulmonary fibrosis than in those who had mild–moderate disease. As is known, one of the possible complications of ARDS is pulmonary fibrosis [35,47]. The risk of developing pulmonary fibrosis is related to the cellular mechanisms that occur in response to acute lung injury and can lead to abnormal and persistent inflammatory response and excessive proliferation of fibroblasts. McGroder et al. evaluated 76 patients at 4 months after hospitalization. Twenty percent of non-mechanically ventilated and 72% of mechanically ventilated patients had fibrotic-like abnormalities (reticulations, traction bronchiectasis or honeycombing) at high-resolution chest CT scan [48]. These abnormalities were correlated with decrements in lung function, cough and frailty but not with dyspnea. Furthermore, this study identified severity of initial illness, duration of mechanical ventilation, the lactate dehydrogenase levels on admission and leukocyte telomere length as independent risk factors for the development of fibrotic-like abnormalities [48]. In a prospective study reporting respiratory outcomes at 12 months after discharge in people recovered from severe COVID-19 who did not require mechanical ventilation, 24% of patients had radiological abnormalities including interstitial thickening and reticular opacity, potential signs of evolving fibrosis [49].

One of the many unanswered questions about post-COVID-19 pulmonary fibrosis patients is whether there is a prompt therapy that may avoid potentially permanent lung damage. In a study by Myall et al., at one month after discharge, in 30 symptomatic patients with evidence of interstitial lung disease (mainly organizing pneumonia) and significant functional deficit, steroid treatment (initial dose of 0.5 mg/kg prednisolone, weaned over 3 weeks) was associated with significant symptomatic, functional and radiological improvement [50]. However, few data have been published on this topic and further studies are needed to clarify how to treat these patients.

Specifically, steroids alone do not seem to be enough to avoid the development of fibrosis [51]. Nevertheless, it should be stated that there is not a consensus on the use of anti-fibrotics in the prevention and arresting of lung fibrosis in COVID-19 survivors yet. Nevertheless, there is a strong rationale for their potential usefulness [52]. They could be reserved for some groups of COVID-19 patients, such as the most severe ARDS cases that are most likely to end up with fibrosis [53].

Anti-fibrotic drugs, such as pirfenidone and nintedanib, have anti-inflammatory effects as well and thus they may be used even in the acute phase of COVID-19 pneumonia [54]; however, there are a few concerns regarding anti-fibrotics in the acute phase. Many COVID-19 patients have hepatic dysfunction, but the anti-fibrotics pirfenidone and nintedanib cause hepatotoxicity [55]. Furthermore, nintedanib is associated with amplified risk of bleeding as most COVID-19 patients are on anticoagulants [55].

Specifically, the use of these drugs in COVID-19 patients is currently being studied in some trials (NCT04856111 and NCT04653831). Moreover, blocking CD147 could also prevent pulmonary fibrosis caused by COVID-19 [56].

Regarding non-pharmacological treatment, in December 2020, the ERS/ATS task force strongly recommended early, bedside rehabilitation for patients affected by severe COVID-19 and suggested a comprehensive pulmonary rehabilitation program for COVID-19 survivors with pre-existing/ongoing lung function impairment at 6–8 weeks following hospital discharge [57].

In conclusion, the management of the respiratory sequelae of COVID-19 should consider clinical, functional and radiological aspects. Since the course of the abnormalities described above is not known, careful monitoring of the disease in survivors is required.

## 3. Cardiovascular Involvement

**Figure 1:**
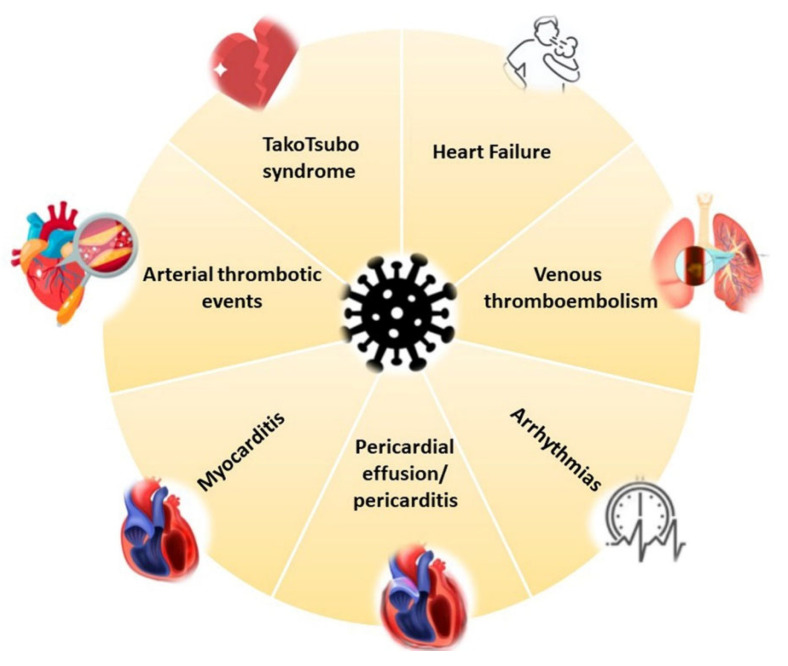
COVID-19 cardiovascular involvement.

**Figure 2:**
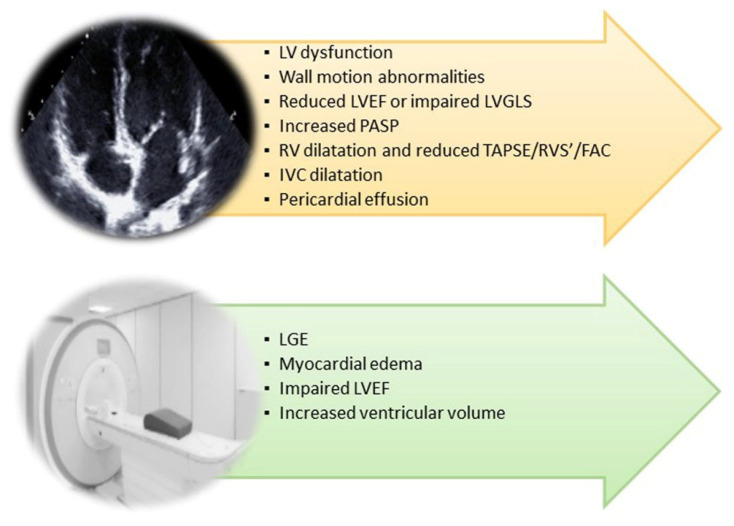
Cardiac imaging techniques’ main findings in post-COVID-19 syndrome. FAC: Fractional Area Change; IVC: Inferior Vena Cava; LGE: Late Gadolinium Enhancement; LV: Left Ventricular; LVEF: Left Ventricular Ejection Fraction; PASP: Pulmonary Artery Systolic Pressure; RVS’: TDI of Tricuspid Annulus; TAPSE: Tricuspid Annular Plane Systolic Excursion.

COVID-19 affects the CV system in the acute phase, but heart complications can also arise during the post-recovery phase [4]. Specifically, reports of myocardial damage in association with COVID-19 comprise acute ischemic injury (type 1 myocardial infarction) [58], along with non-ischemic injury (i.e., myocarditis) [59,60], stress cardiomyopathy [61], heart failure (HF) [62] and secondary cardiac injury caused by sepsis and critical illness [63] (Figure 1 and Figure 2).

Mechanisms of myocardial injury may be indirect via systemic inflammatory response or direct (viral infection, thought to be less common) [64]. Specifically, autopsy studies on 39 COVID-19 patients identified virus in the heart tissue of 62.5% of patients [65]. The following inflammatory response may lead to cardiomyocyte death and fibro-fatty displacement of desmosomal proteins [66]. Recovered patients may have persistently increased cardiometabolic demand, as shown in long-term evaluation of SARS survivors [67], due to the reduced cardiac reserve, corticosteroid use and dysregulation of the renin–angiotensin–aldosterone system (RAAS).

Angiotensin converting enzyme 2 (ACE2) plays a crucial role in the development of CV complications [68]. Specifically, high expression of ACE2 in COVID-19 patients leads to an RAAS overactivation, with consequent dysregulation of electrolytes and fluid homeostasis [68]. Thus, excessive vasoconstriction and blood flow acceleration augment the risk of thrombosis and hypertension [69]. Moreover, high blood pressure increases the afterload on the heart and subsequently causes organic pathological changes such as cardiac dilation [70]. Myocardial fibrosis or scarring, and resultant cardiomyopathy from viral infection, can produce arrhythmias [71].

The type of acute cardiac damage that COVID-19 patients have remains uncertain. Nevertheless, there is evidence that heart attack-like events are responsible and, consequently, randomizing patients to cardioprotective medicines (NCT04333407) will help us understand the role of the CV system in COVID-19 disease. Moreover, bromodomain and extraterminal family inhibitors (BETis) improved dysfunction in human cardiac organoids (hCOs) and totally avoided cardiac dysfunction and death in a mouse cytokine storm model [72]. Furthermore, a BETi decreases transcription of genes in the viral response, reduces ACE2 expression and decreases SARS-CoV-2 infection of cardiomyocytes [72]. Together, BETis, including apabetalone, are encouraging candidates to prevent COVID-19 cardiac damage [72,73].

Palpitations and chest pain are the most common subjective findings [9]. A study by Frankfurt University Hospital revealed that 78% of survivors of COVID-19 had CV alterations, and 60% of them still showed signs of persistent myocardial inflammation more than two months after the diagnosis [74]. The results propose that long-term sequelae, for example, arrhythmias and HF, are also probable in apparently healthy people [75].

Furthermore, a study from Wuhan, China revealed that about 20% of COVID-19 patients had CV damage and the patients’ conditions would worsen if their IL-6 levels were high [76,77]. Specifically, the most severe CV complication in COVID-19 is myocarditis [78].

Myocardial damage could be the cause of an inflammatory cascade and following fibrosis; moreover, the distribution and extent of this inflammatory reaction could result in unfavorable ventricular remodeling and arrhythmias. Radin et al. showed that COVID-19 patients had prolonged relative tachycardia that lasted on average 79 days after symptom onset; specifically, 13.7% of patients did not return to resting heart rate baseline until after 133 days [79]. Furthermore, those hospitalized are at risk of even more severe sequelae, such as HF, arrhythmias, myocardial infarction and stroke (three times greater than matched controls patients) [80].

Likewise, other complications have been reported, such as postural orthostatic tachycardia syndrome [81,82] and orthostatic intolerance without hemodynamic effects [83]. Lastly, right ventricular dysfunction in response to fibrotic lung injury, pulmonary hypertension and/or clot burden in patients recovering from severe disease have also been described with an incidence of diastolic dysfunction of 32–55%, and an occurrence of pulmonary hypertension of 10–35% up to 12 weeks following the acute phase [84,85,86].

Existing data show that prolonged CV symptoms can be expected in a large proportion of COVID-19 patients even in the long term; consequently, numerous studies are presently being conducted to find out the long-term repercussions of COVID-19, such as the CV-COVID-19 registry (NCT04359927), which aims to determine the frequency of clinically important endpoints such as CV mortality, acute coronary syndrome, pulmonary embolism and hospital admission due to HF [87].

## 4. Brain Involvement

Brain involvement of COVID-19 generally arises during the acute phase of the infection. However, neurological and psychiatric sequelae are also frequent during the post-COVID-19 phase. Specifically, post-COVID-19 neurological syndrome (PCNS) is a side effect of COVID-19 that is increasingly recognized; consequently, cognitive and psychiatric functions need strict monitoring in COVID-19 patients who survive beyond the acute phase [88,89,90]. Particularly, heterogeneous symptoms have been described, however, the commonest are muscle pain, dizziness, headaches, fatigue, anosmia, amnestic dysfunction, dysexecutive syndrome, ataxia, tetraparesis and sleep disorders [29,91].

Rehabilitation is recommended to avoid long-term neurological complications [92]; however, standard mobility programs are challenging for those who are in an ICU. Consequently, the aim of the ongoing trial NCT04685213 is to test the feasibility and effectiveness of daily use of lower extremity electrical stimulation therapy, as a practical solution to retain lower extremity muscle mass.

The pathogenetic mechanisms responsible for consequences for the nervous system are still unknown, although different potential pathways could be involved.

**Figure 3:**
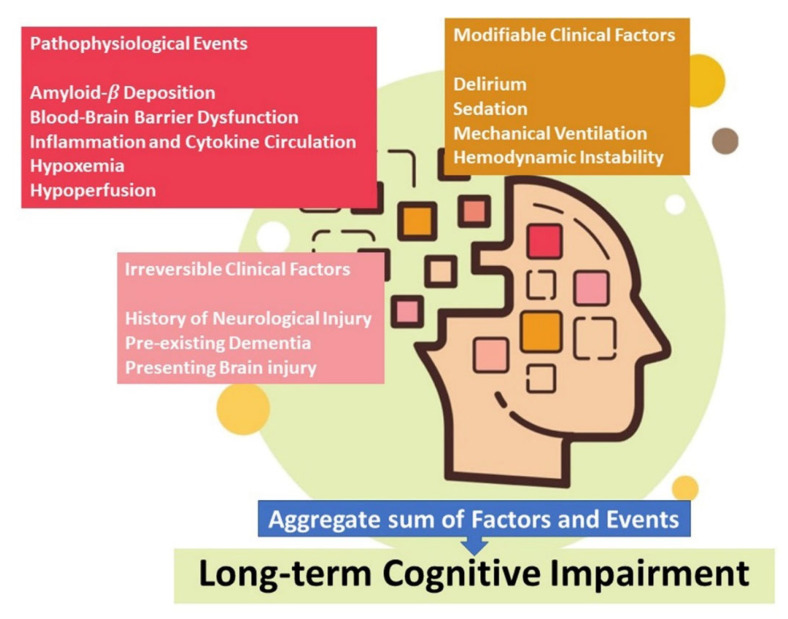
Mechanism of nervous system damage in post-COVID-19 syndrome.

Firstly, systemic inflammation could increase the velocity of the evolution of neurodegenerative processes aggravating clinical signs and symptoms of neurological disease already present at the time of the infection (Figure 3).

Specifically, inflammation determines endothelial alterations and migration of leukocytes across the blood–brain barrier [93]. A second possible pathway involves direct damage of SARS-CoV-2 to cerebral tissue. Specifically, this mechanism involves transsynaptic transfer across infected neurons, through nerves (e.g., olfactory), with a possible role of the interaction between ACE2 receptors and spike proteins of the virus [94,95]. Although the analysis of histological cerebral pieces demonstrates SARS-CoV-2 RNA and proteins in the brains of patients affected by COVID-19, factors such as cytokine storm, neuroimmune stimulation, systemic SARS-CoV-2 infection and direct damage caused by the virus may coexist [96].

Furthermore, COVID-19 has long-term effects on mood: one third of COVID-19 patients present depressive symptoms or clinically significant depression. Particularly, it is noticed that these symptoms develop more commonly during the first 12 weeks after the infection [97]. Accordingly, a study by Hai-Xin Bo et al., involving 714 COVID-19 patients, gave evidence that nearly 97% of the patients developed symptoms compatible with post-traumatic stress disorders (PTSDs). Nevertheless, COVID-19 patients often experience long quarantine isolation, so anxiety, decreased mood and insomnia commonly arise with a worsening of the quality of life [98].

The biochemical substrate of depression related to COVID-19 is systemic inflammation. Particularly, cytokine storm and increased inflammatory factors contribute to the augmentation of the permeability of the blood–brain barrier. The effect is a reduction in tryptophan and serotonin circulating levels and an augmentation of toxics such as kynurenine, quinolinic acid or 3-hydroxykynurenine [99]. These variations facilitate neurotoxicity, neurodegeneration and reduction in neurogenesis and synaptic plasticity [99].

## 5. Organ Crosstalk and Management of Post-COVID Conditions

We are progressively assisting the passage of COVID-19 from an acute disease to a chronic one. SARS-CoV-2 is a virus determining a systemic disease, whose sequelae involve the main vital organs such as the lung, heart and brain, with often reciprocal crosstalk and influence.

Protracted physical symptoms after COVID-19, including CV and respiratory symptoms, are closely related to a higher probability of developing psychiatric diseases [100,101,102]. Furthermore, post-COVID-19 syndrome might be experienced more strongly by depressed patients, or these dimensions might involve shared factors such as a high level of neuroticism (i.e., tendency to experience negative emotions such as anger, fear or sadness with limited tolerance for aversive stimuli) [103,104]. On the other hand, a rise in the incidence of Takotsubo syndrome seems to be an effect of the COVID-19 pandemic, with the incidence of Takotsubo syndrome rising 4.5-fold during the pandemic even in people without severe acute respiratory syndrome [105]. Specifically, studies show that chronic anxiety and/or depression are typical in patients with Takotsubo syndrome [105].

Interrupted sleep and irregular sleep patterns are frequent in COVID-19 patients [106]; either can lead to impaired autonomic tone and endothelial vasomotor dysfunction [107] with related symptoms. Moreover, depression affects the quality of life, habits and, consequently, the health status in its complexity. A modification of lifestyle causes, in the long term, an increased risk of CV events and possibly increased blood pressure [108]. Additionally, memory problems can increase CV risk, for example, if the patient forgets to take medication on time.

Circulatory impairment, due to persistent myocardial inflammation and associated altered cardiac function, also includes reduced lung perfusion, which may be relevant in generating dyspnea [109]. Moreover, COVID-19 infection has been associated with significant clot formation such as pulmonary emboli; consequently, it is possible that some patients may develop chronic thromboembolic pulmonary hypertension with dyspnea. Moreover, post-COVID-19 pulmonary fibrosis can lead to right HF.

**Figure 4:**
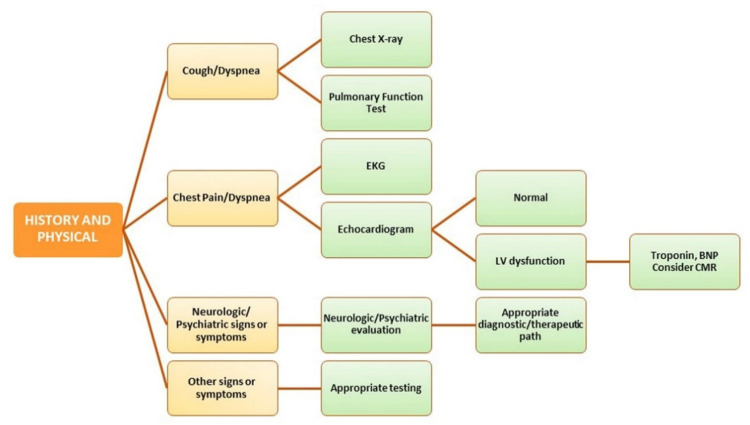
Management of post-COVID-19 syndrome. BNP: Brain Natriuretic Peptide; CMR: Cardiovascular Magnetic Resonance; EKG: Electrocardiogram; LV: Left Ventricular.

Therefore, treatment and management of patients with long COVID require a multidisciplinary approach. Mild to severe symptoms must be adequately evaluated according to the underlying diagnosis (Figure 4).

Specifically, treatment options can range from symptomatic medications to rehabilitation physiotherapy, psychological support and standard major disease protocols such as those for secondary bacterial infections, pulmonary embolism or cardio- and cerebrovascular accidents [110].

Cardio-pulmonary and neurological rehabilitation associated with early mobilization are of great utility in patients with post-intensive care syndrome [111,112]. Mental health support in long COVID patients with onset of coping issues, anxiety, depression and PTSD requires special management [113,114].

In consideration of the various types of possible sequelae and the lack of knowledge regarding COVID-19’s long-term effects, evaluating treatment options is very challenging. The difficulty also consists of the management of previous underlying comorbidities such as hypertension, diabetes and chronic obstructive pulmonary disease, which can worsen during COVID-19 and require new optimized treatment [115].

The duration and frequency of follow-up can drastically vary. Recommendations suggest a minimum of 12 months’ follow-up, with seven healthcare professional visits (at least four in person) accompanied by blood tests, 6-min walking tests (6MWTs) and thoracic radiological examinations [116]. Patients with severe COVID-19 complications will inevitably need more intensive in-person follow-ups, while mild–moderate patients could be managed with more telemedicine and fewer face-to-face consultations. Management strategies for long COVID could benefit from regulated patient-reported outcomes and harnessing digital medical technologies [117,118]. The clinical course of each patient is unique and follow-up must be managed accordingly.

The implementation of guidelines for long COVID management is essential. Fortunately, some countries have started implementing clinical guidelines to help clinicians [119]. The healthcare system will go through economic, organizational and structural difficulties as this disease continues to spread and more and more people will experience chronic persistence of COVID-19 symptoms. In the near future, the overload of healthcare support will greatly benefit from a well-defined long COVID management protocol.

## 6. Conclusions

Since the beginning of the COVID-19 pandemic, the respiratory, CV and nervous systems have been closely linked to this disease. An emerging problem in the post-epidemic era is the sequelae of COVID-19. Long COVID is an important health issue affecting the general population worldwide; however, to date, researchers have focused on the ongoing active phase of the disease. The damage in post-COVID-19 syndrome seems to be multifactorial, with a key role in impaired regulation of the RAAS, inflammation and coagulopathy disorders. A robust investigation on understanding all the aspects related to long-term COVID-19’s consequences is important to identify the risk factors and etiology of the long-lasting deleterious effects of COVID-19 to improve prevention, rehabilitation, clinical/public health management and long-term COVID-19 outcomes.

## Figures and Tables

**Table 1:** Reported cardiovascular, respiratory and nervous post-COVID-19 complications.

System Involved	Symptoms	Monitoring System
Cardiovascular complications	↑ Resting rates Palpitation Elevation in the blood pressure Pericardial chest pain Chest tightness T2 signal and positive LGE Myocardial edema Pericardial effusion Diastolic dysfunction Pulmonary hypertension Non-specific patterns of capillary abnormalities Hemosiderin deposits Cardiac arrhythmias	Echocardiogram Electrocardiogram CMR
Respiratory complications	Breathlessness/dyspnea/tachypnea Cough Lung function abnormalities (↓ FEV1, ↓ FEV1/FVC) Pulmonary fibrosis Interstitial thickening crazy paving Residual ground-glass opacity Abnormal diffusion Pulmonary embolism Pneumonia	Pulse oximetry 6MWT PFTs Chest X-ray High-resolution computed tomography of the chest Computed tomography pulmonary angiogram
Nervous system complications	Post-traumatic stress disorder Depression or anxiety Memory problems Insomnia Sleeping disturbance Cognitive impairment and concentration problem Stigma Headaches Muscle weakness Dizziness Critical illness neuropathy Residual smelling disorder Acute inflammatory demyelinating polyradiculopathy	Standard screening tools

CMR: Cardiovascular Magnetic Resonance; FEV1: Forced Expiratory Volume 1; FVC: Forced Vital Capacity; LGE: Late Gadolinium Enhancement; 6MWT: Six-Minute Walking Test; PFTs: Pulmonary Function Tests; ↑: increased; ↓: decreased.
